# COVID-19 Guidelines to Protect Healthcare Workers at Hospitals and Dental Professionals at Dental Office

**DOI:** 10.4314/ejhs.v30i6.23

**Published:** 2020-11

**Authors:** Bassel Tarakji, Mohammad Zakaria Nassani, Faisal Mehsen Alali, Abdulwahab A Abuderman

**Affiliations:** 1 Department of Oral and Maxillofacial Surgery and Diagnostic Sciences, Prince Sattam Bin Abdulaziz University, College of Dentistry, Al Kharj, Saudi Arabi; 2 Department of Restorative and Prosthetic Dental Sciences, College of Dentistry, Dar Al Uloom University, Riyadh, Saudi Arabia; 3 Department of Oral and Maxillofacial Surgery and Diagnostic Sciences, Prince Sattam Bin Abdulaziz University, College of Dentistry, Al Kharj, Saudi Arabi; 4 Department of Basic Medical Sciences, Prince Sattam Bin Abdulaziz University, College of Medicine, Al Kharj, Saudi Arabi

**Keywords:** CVID-19, health worker at hospitals, infection control, dental professionals, personal protective equipment

## Abstract

**Background:**

Coronavirus disease 2019 is an infectious disease caused by severe acute respiratory syndrome coronavirus 2. This study aimed to address the preventive procedures to protect healthcare workers at hospital to avoid COVID-19, and infection control procedures to protect dental professionals in dental office.

**Methods:**

We conducted a search of published articles from PubMed, google scholar databases using key words such as COVID-19, healthcare worker, infection control, and dental practice. Relevant articles were identified and reviewed. Most published papers were clinical reports and case studies. We have selected some of the current published papers written in English in 2020.

**Results:**

Infection control procedures to protect health workers at hospitals, and dental professionals at dental office were summurised and presented. Infection control procedures for healthcare workers at hospitals include Personal protective equipment, Korea filter (KF)94 respirator, goggles, face protector, disposable waterproof long-arm gown, and gloves, and others. Extra-protection procedures should be taken with old and vulnerable healthcare workers. Dental professionals should evaluate patients in advance before starting dental treatment. Aerosols generating procedures should be avoided and personal protective equipment should be used. Dental treatment should be restricted to emergency cases only.

**Conclusion:**

Old medical staff should be in safer distance to avoid infection, but young physicians and nurses should work at frontline as their immunity is better than their colleagues at old age. Screening patients and measurement of the body temperature are essential measures before dental treatment.

## Introduction

COVID-19 has become a health disaster for human beings worldwide (1). Nowadays, the number of infected persons is in dramatic daily rise. Most people all over the world are staying at home to avoid threat of exposure to this invisible virus, and a feeling of fear and uncertainty has become the general mode. The risk of cross infection between healthcare workers (HCW) in hospitals, dental professionals and patients is quite high (1–2). Unfortunately, there is no consensus on international infection control protocol for the safety of (HCW), dental professionals and their patients. This short communication tries to provide recommendations or guidelines for both (HCW) in hospitals and dental care providers to avoid infection by COVID19. The healthcare workers in hospitals work to fight at the first line to save lives of patients infected with COVID-19, and dental professionals are working in a risky environment to provide dental treatment services.

## Methods

A search of published articles from PubMed, google scholar databases using keywords such as COVID-19, healthcare worker, infection control and dental practice was undertaken. Relevant papers regarding COVID-19, healthcare workers, infection control, and dental practice were reviewed and included in this article.

## Results

The selected papers comprised the following important points: problems, difficulties, and infection control procedures to protect healthcare workers in hospitals, personal protective equipment (PPE) and social distance. Young medical staff should work in frontline to fight COVID-19 at hospital. Screening room and body temperature, avoid of aerosols, and treatment of emergency cases are important elements to avoid COVID-19.

## Discussion

Our short communication discussed the following headings:

**Problems, difficulties and infection control procedures to protect healthcare workers in hospitals**: Healthcare workers are facing heavy workloads and high risk of infection with COVID-19. On February 20, 2020, 2055 confirmed cases were detected in China (3). The safety of HCW is essential in order for them to provide the best possible medical services for infected persons. The number of infected cases increased daily in some countries, and doctors and nurses worked for 12 hours a day (4). The increased workload and shortage of HCW will increase the possibility of medical staff to be infected with COVID-19 (4). Personal protective equipment is essential to protect HCW in hospitals. Some useful instructions related to personal protective equipment (PPE) including hair should be fixed and jewelry should be removed to avoid contamination. Medical staff should go to bathroom before start working, therefore it is recommended to drink water before using (PPE) to avoid dehydration. (4). In case of damage or contamination of PPE, PPE should be replaced. The guideline for the care of severe patients with COVID-19 infection reported by the Korean Society of Critical Care Medicine including: HCWs should wear personal protective equipment, such as a Korea filter (KF)94 respirator, goggles, face protector, disposable waterproof long-arm gown, and gloves (4–5).

There are some daily medical procedures that should be taken into consideration for HCW. In case of tracheal intubation, laryngoscopy and cardiopulmonary resuscitation, personal protective equipment including a KF94 respirator, goggles, or face protector, wholebody protective clothing and gloves are required (4–5). It is preferred that at least 1 physician and 1 assistant nurse wear a powered air-purifying respirator (4). For protection of HCWs from droplet-transmitted infections, the National Institute for Occupational Safety and Health recommended the use of N95 respirator than surgical masks (6). It is recommended to increase training for HCWs and follow the instructions of safety and antiseptic techniques. By doing so the risk of transmission of COVID-19 in hospital will be lowered (4–5). Healthcare workers collect daily the specimens from the nasopharynx, oropharynx and sputum for suspected and infected COVID-19. Therefore, continuous courses of the infection control and education will help HCWs to avoid COVID19 (4–5). The majority of HCWs stay at hospital all the times due to their fear of transmitting the infection to their family members if they become infected (4–5). Most HCWs are suffering from psychological stress due to workload and fear of being infected with COVID-19. Therefore, the continuous communication with HCWs will reduce this stress (4–7). Regular test of COVID-19 for healthcare workers should be recommended. The daily use of personal protective equipment for long hours will cause eczema (4). Ointments and placing adhesive bandage on the skin are preventive methods (4). The physical safety, psychological stability and infection control procedures are important factors for healthcare workers at hospital (4–7).

**Protect older and vulnerable health care workers from Covid-19**: We address in this short communication study an interesting point regarding the extra-protection procedures for old and vulnerable healthcare workers. This group of healthcare workers is not highlighted in the literature. Dr. Aaron Kofman, and Alfonso Hernandez-Romieu (8) reported that 4800, 3300 healthcare workers were infected with COVID-19 in China, and Italy respectively. Health authorities of hospitals should establish policies of social distancing to protect oldest and vulnerable healthcare workers as most of HCWs are working together and with contact of infected COVID-19 patients, so that they are at risk to be infected from the patients they take care of (8). In Italy (8), death rates from Covid-19 were three times higher for those between the ages of 50 and 59, and 10 times higher for those between the ages of 60 and 69, compared to those between the ages of 30 and 50. The severe cases or death from Covid-19 was detected among older adults and those with chronic medical conditions (8). Most consultants, senior physicians and experienced nurses are susceptible to infection with COVID-19 due to old age and chronic diseases such as hypertension or diabetes, immunosuppressant and heart disease (1,8). On 2018, there were 512,000 physicians over the age of 50 in America, representing just over half of the entire U.S.A's physician workforce. The average age of nurses in the U.S.A is 51 years (8). Some healthcare institutions (8) decided to ask HCWs that might be at high risk from COVID-19 to take an early vacation. Other institutions allow for health care workers to continue their work on wards if they do not have health problems such as pregnancy, or chronic disease (8). Older and vulnerable healthcare workers can treat infected COVID-19 patients from safer distance than they do today. In USA, they shifted HCWs to virtual meetings and phone conversations (8). Young healthcare workers have better immunity than the old to absorb this load in wards, but old senior health workers can have rounds in hospital if needed by keeping social distance to guide the young physician and nurses by checking electronic medical records and receiving information directly from residents and interns (8). We believe that young health workers can take responsibilities to be in the frontline to fight COVID-19 to protect their older colleagues from this risk. Senior physicians can provide good care for their patients from safer distance but not in frontline. The existence of virtual tools such as telemedicine and social media can facilitate the communication between young and old health workers. We recommend the health authorities to establish the policies to provide fast training for young and interns staff to protect old health workers and avoid collapse in health systems.

**Protection dental professionals from COVID19**: First of all, we believe that sound knowledge among dental practitioners/dental care providers of routes of transmission, incubation period, virus behavior, clinical manifestations, diagnostic tools, and extraprotection procedures is essential to minimize the risk of infection with COVID-19 in the dental office. It has, for example, to be clear for the dental professionals that contact with contaminated instruments and environmental surfaces has a major role in transmission of the COVID-19 (9). Peng et al. (9) indicated that in dental clinics SARS-CoV, and Middle East Respiratory Syndrome Coronavirus (MERS-CoV), can stay on surfaces such as glass, for few days. Also, the World Health Organization (WHO) reported that COVID-19 can stay on surfaces from few hours to several days (10). Moreover, the available literature shows that COVID-19 has the ability to spread through droplets and aerosols from infected patients. Also, it can spread through contact with saliva and conjunctival, nasal or oral mucosa (9–10). What is worse, COVID-19 is a tricky virus with long incubation period of up to 14 days.

In principle, the dental practitioner should not treat subjects infected with COVID-19. Referral of such subjects to a specialized hospital is the recommended action. Besides, dental treatment of patients attending the dental office with symptoms of acute respiratory infections should be avoided. However, symptoms may be absent and as a preventive measure, all patients visiting the dental office should first undergo strict screening for body temperature to investigate any signs for a fever. Special and isolated screening clinic is highly recommended. In the screening room/clinic, the patient should be invited to complete a questionnaire that seeks information regarding any fever, sneezing, cough and other respiratory problems that occurred in the last 14 days. Also, questions related to recent travel history and meeting with people who are infected with COVID-19 should be answered. In case of a positive answer to any of the listed questions, the dental treatment should be postponed at least for 14 days. On the other hand, dental treatment can be undertaken if the answers to the former questions were negative. However, in case of undertaking dental treatment at the time of COVID-19 eruption, strict measures of protection and infection control should be followed including avoidance the use of aerosols generating devices and droplets (9). Under the pandemic circumstances of spread of COVID-19, any dental intervention should be kept minimum and restricted to emergency cases. Among the precautionary measures is hand hygiene. The Infection Control Department of the West China Hospital of Stomatology at Sichuan University indicated the importance of hand hygiene and recommended frequent handwashing before and after any dental procedure (9). Also, dental professionals should avoid touching their noses, mouths and eyes. Peng et al. (9) reported that airborne droplet transmission of infection is the main way of infection spread in the dental clinic. The authors strongly recommended the use of barrier-protection disposal equipment, including protective eyewear, masks such as N95 respirators, gloves, caps, face shields and protective outerwear. Moreover, dentists may require the patient to rinse with chlorhexidine mouthwash, or mouthwash containing oxidative materials to decrease the salivary load of oral microbes and COVID-19 especially in cases when rubber dam is not used. Also, antirestrictive valves dental handpieces should be used to avoid cross infection of COVID-19 (9). An important point is related to the environment of the dental clinic that should be cleaned and disinfected meticulously after each patient due to the long existing time of COVID-19 on surfaces (9–11). Again, all health workers in the dental clinic including dentists, nurses, hygienists and others should always follow firm protection measures all the times. Although such strict extra protection procedures will increase the cost on the dental office, we need to remember that prevention is more cost-effective compared to the burden of infection with COVID-19 on individuals and the whole community.

Based on a study conducted by Gazal (2020) (12), diabetic patients with fasting blood glucose level of ≥ 240 mg/dl (13.3 mmol/l) or random blood glucose level of ≥234 mg/dl (13 mmol/l) should be denied any dental treatment because they are at high risk of coronavirus infection. Patients with uncontrolled diabetes are considered immunosuppressed due to the negative effects of elevated blood sugars on the immune system. Immune defects arise from the macrophages which lose its appetite and cause a slowing down in the process of phagocytosis. High blood glucose levels reduce the secretion of powerful vasodilator nitric oxide (NO) in the circulatory system, cause stiffening and narrowing of blood vessels. Thus, uncontrolled diabetic patients are associated with poor circulation. Changing room for medical and dental practitioners should be modified in a way that allows the practitioner to enter through a specific door for the clinics and, upon completion of work, they go to the changing room from a second entrance so that we avoid any possibility even if it is small for the transmission of infection.

In conclusion, senior old healthcare workers at hospitals should work in safer distance not in frontline to avoid infection with COVID-19. Personal protective equipment is essential for both healthcare workers at hospital and dental professionals. We believe that the aforementioned recommendations may be useful guidance for healthcare workers and dental professionals at the time of such global health crisis.

## References

[1] Phelan AL, Katz R, Gostin LO (2020). The novel coronavirus originating in Wuhan, China: Challenges for global health governance. JAMA.

[2] Meng L, Hua F, Bian Z (2020). Coronavirus disease 2019 (COVID-19): Emerging and future challenges for dental and oral Medicine. J Dent Res.

[3] WHO (2020). Report of the WHO-China Joint Mission on Coronavirus Disease 2019 (COVID-19).

[4] Sun Huh (2020). How to train health personnel to protect themselves from SARS-CoV-2 (novel coronavirus) infection when caring for a patient or suspected case. J Educ Eval Health Prof.

[5] Korea Centers for Diseases and Prevention (2020). Board for coronavirus infection (COVID-19) [Internet].

[6] MacIntyre CR, Chughtai AA, Rahman B (2017). The efficacy of medical masks and respirators against respiratory infection in healthcare workers. Influenza Other Respir Viruses.

[7] Chou R, Dana T, Buckley DI, Selph S, Fu R, Totten A M (2020). Epidemiology of and Risk Factors for Coron avirus Infection in Health Care Workers. Ann Intern Med.

[8] https://www.statnews.com/2020/03/25/protect-older-and-vulnerable-health-careworkers-from-covid-19.

[9] Peng X, Xu X, Li Y, Cheng L, Zhou X, Ren B (2020). Transmission routes of 2019-nCoV and controls in dental practice. Int J Oral Sci.

[10] World Health Organization (2020). COVID-19 Questions and answers.

[11] Chen J (2020). Pathogenicity and transmissibility of 2019-nCoV-A quick overview and comparison with other emerging viruses. Microb Infect.

[12] Gazal G (2020). Management of an emergency tooth extraction in diabetic patients on the dental chair. Saudi Dent J.

